# Book Reviews

**Published:** 1897-08

**Authors:** 


### Book Reviews.

AUTOSCOPY OF THE LARYNX AND TRACHÆ. By Alfred Kirstein, M. D. Published by the F. O. Davis Co., Philadelphia.

This little monograph is a translation from the German by Dr. Max Thorner, of Cincinnati. The title indicates the subject treated that is the direct inspection of the larynx and teaches without the aid of the laryngoscopic mirrors. The word autoscopy certainly does not convey the author's meaning. While we have not seen this method used, experience in the examination of throats would certainly teach us that only in exceptional cases could it be used. The author himself admits as much. In cases where we have a subject with a large throat, whose gagging reflexes are but slight, and who does not care what manipulations are instituted in these parts, then autoscopy could probably be used. But such patients rarely have throat trouble, unless it be some kind of growth. Usually throat troubles belong just to the opposite class of cases. However, there is no doubt of the utility of this method when we wish to remove growths from the larynx; for operating by a mirror picture with the position of the parts reversed is sometimes a little annoying. Certainly the author is to be praised for introducing the method and bringing it so prominently before the profession. It is a subject well worthy the consideration of all workers in laryngology.

DUNBAR ROY.

---

THE DISEASES OF THE STOMACH. By Dr. C. A. Ewald, Extraordinary Professor of Medicine at the University of Berlin, etc. Translated and edited with numerous additions from the third German edition. By Morris Manges, A. M., M. D. Second Revised Edition, New York, D. Appleton & Co., New York. 1897.

Only commendation can be given this translation of the third edition of Prof. Ewald's work, as its high character is acknowledged as well in this country as in Europe. The subject is clearly and fully treated in accordance with the latest scientific views. The translator adds to the text all facts of interest brought forward since the German edition was published, thus bringing the work up to date. In these days when the field of the general practitioner is being so greatly limited by specialists, it becomes necessary for every physician to keep informed in regard to the advances being made both in diagnosis and treatment in this important branch, lest it too be lost to him. For this reason, if for no other, this work should be in the library of every general practitioner. We can most heartily commend it.

EYF STRAIN IN HEALTH AND DISEASE. By A. L. Ranney, M. D,, New York, Published by the F. A. Davis Company, Philadelphia.

To those who are familiar with the discussions recently waged by Dr. Ranney in the *New York Medical Journal* upon the treatment of epilepsy by graduated tenotomies of the extra ocular muscles, the character of this work will be readily surmised. Dr. Ranney has certainly succeeded in making his book most attractive in style, and its contents certainly remind one of the wizard writings of the middle ages. It would seem as if the "golden egg" had been found, that henceforth all ills can be banished from the human frame. Dr. Ranney from his writings would certainly lead one to consider him a most deeply-dyed enthusiast, although it is over a subject well worthy the investigations of any scientific mind. We have long since found out that in "every extreme measure there is a germ of truth," and the subject of this brother is no exception to the rule. We are living in an advanced and scientific age, and the physician who adheres to the old remedial of diagnosis because forsooth he is perfectly well satisfied with what he learnt twenty years ago, or because theoretically he sees no good in these new innovations, must certainly fall by the wayside and be looked upon as a relic of bygone ages. While we do not fully agree with the author in all of his ideas in regard to the usual dependence of various ailments of the body upon eye-strain, yet there are a great many things therein contained which are worthy of thought, and therefore worthy of practice. The subject with which Dr. Ranney deals is a difficult one, and the more you study, the more intricate does it become. The author's views, as set forth in his book, are these:

He holds that all functional nerves of the body (if they can be so-called), such as headache, insomnia, chorea, epilepsy, nervous prostration, etc., in their last analysis can be shown in nearly all cases to be dependent upon some form of eye-strain. By "eye-strain," he means either the presence of some error of refraction such as hyperopia, myopia, astigmatism, or more especially some error in the muscular equilibrium of the eye, such as a tendency to turning in (esphoria), turning out (exophoria), or turning up or down (hyperphoria.) His results as exemplified in the cynical cases reported certainly read like a fairy tale. That the profession at large will not accept Dr. Ranney's deductions is also true. As for the writer of this criticism, who is no neurologist, his own experience has taught him that there is a great deal in the lack of muscular equilibrium as the cause of headaches, and if this be true, then there is no reason why other nervous manifestations should not have a similar cause. We would never consider that any patient's eyes had been thoroughly examined and tested until the state of the muscles had been ascertained by our modern methods of diagnosis. One of the chief obstructions which lie in the way of further scientific development of this subject is the fact that it opens up a field to unskilled and unscientific physicians, who, by an ignorance put into practice, will bring disrepute upon the subject. The cutting of muscles is an easy thing, but when to cut is a different matter. This point is well exemplified in a note received by Dr. Ranney from a physician, which reads as follows: "What troubles me more is the fact that I am not sufficiently posted on the diagnosis of the eye to use your valuable information. I am very much inclined, however, to go it blind on the case I spoke of and give it a trial." We can not of course agree with Dr. Ranney in all his views. In the first place, because we do regard him as too much of an enthusiast, and in the second place, because we have seen no clinical demonstration of the facts therein contained. But the oculist who ignores the importance of the eye muscles as a causal factor in many cases of asthenopia and headache, will certainly come to grief as far as his success is concerned.

Experience teaches us that there is a great field for labor and investigation in the study of the muscular anomalies of the eye, and while the Utopian success of Dr. Ranney may never be realized, yet it has done this much, that is, "set us thinking." There is certainly one criticism which we would make and feel justified in so doing, and that is, the fact that due credit has not been given in this book to Dr. Geo. T. Stevens, the pioneer worker, and certainly the most advanced thinker on this subject. One would think by reading the book that Dr. Ranney was the sole originator of these methods for treating and recognizing muscular anomalies of the eye, while in fact he has but carried out the facts as deduced and laid down by Dr. Stevens. Some good men still ridicule the idea that there exists whatever any muscular anomalies except strabisms, and especially the advanced ideas of treatment; yet daily observation but strengthens us in our estimation of the importance of this subject. He is the best physician who decoys no therapeutical measure until he has given it a trial.

Dunbar Roy.

---

Surgical Hints for the Surgeon and General Practitioner. By Howard Lilienthal, M. D., New York. International Journal of Surgery Co. 1897.

Much of the contents of this little book has already appeared in the *International Journal of Surgery* and has been widely copied. The hints cover all branches of surgery and contain much valuable advice and caution in very condensed form. The little work, which is a gem in type and binding, will without doubt be appreciatively read by a large class of surgical workers.

WARNER'S POCKET MEDICAL DICTIONARY. Wm. R. Warner & Co., Philadelphia.

A very handy little volume filled with useful information in the most condensed form available.

---

ANATOMY, DESCRIPTIVE AND SURGICAL. By H. Gray, F. R. S., Fellow of the Royal College of Surgeons; Lecturer on Anatomy at St. George's Hospital Medical College. A new edition, thoroughly revised by American authorities from the thirteenth English edition. Edited by T. Pickering Pick, F. R. C. S., with 772 illustrations, many of which are new. Lea Brothers & Co., Phil. and New York. 1896.

Few books have stood the test of time as has Gray's Anatomy—found in the office of every physician, in the hands of every medical student, upon the dissecting tables of every medical school, it stands a glorious monument to a gifted man.

This revised thirteenth edition, practically a fourteenth edition, by American authorities is a careful revision of the old, with some few sections rewritten and more than one hundred new engravings introduced. To the medical student, Gray's is a necessity, and the practical surgeon will find in its pages, both in word and illustration, that knowledge which will enable him to apply anatomy to the practice of surgery.

---

HYSTERIA AND ALLIED CONDITIONS. George J. Preston. P. Blakiston, Son., & Co., Phila. 1897.

An extremely valuable book for the practitioner of medicine —concise and practical, it deals with the clinical types seen in every-day work—no unneccessary words are used, and after finishing it one can still believe that there are other diseases in the world than hysteria. The author has conscientiously written an instru[illegible]tive text-book rather than a prize thesis.

---

The Medical Gazette Publishing Co., of Cleveland, Ohio, announces a small volume soon to be issued with the title "About Children." The author is Dr. Samuel W. Kelley, of the Cleveland College of Physicians and Surgeons. The book will contain six lectures filled with information for nurses, medical practitioners, students, and all who have the care of children. Advance orders will be filled in September.

---

INTERNATIONAL CLINICS. Vol. I. Seventh series, April, 1897. J. B. Lippincott & Co. Phila., Pa.

This number contains the following subjects:

TREATMENT.

Rules Governing the Treatment of Appendicitis, J. W. White, Phila; Treatment of Syphilis by Hypodermic Injections of Mercury, Prof. Fournier; The Less Common Forms and Modes of Treatment of Syphilitic Affections, T. McCall Anderson; The Treatment of Acute and Chronic Catarrhal Inflammation of Middle Ear, Chas. H. Burnett; The Treatment of Fractures, A. Pierce Gould; Literary Methods in Medicine, W. W. Keen; After Treatment of Abdominal Section, J. M. Baldy; The Treatment of Chronic Retro-displacements of the Uterus, W. Easterly Ashton; The Diagonsis and Treatment of Ulcers of the Cornea, Edward Jackson; Acute Rhinitis and its Treatment, W. E. Casselberry; The Treatment of Psoriasis, W. A. Cantrell; Tuberculosis of the Larynx and its Treatment, W. R. Hoch; Haemoptysis and its Treatment, Thos. J. Mays; The Palliative Treatment of Disease of the Rectum, J. M. Mathews; The Treatment of Pneumonia, John G. Cecil.

MEDICINE.

Milk as an Article of Diet in Disease, Robert Saundby; Enlarged Bronchial Glands with ·Uritral Insufficiency Tuberculosis, Frank C. Wilson; Aortic Aneurism Causing Death by Rupture into the Left Pleural Cavity, Frederick Taylor; The Excessive Secretion of Hydrochloric Acid by the Stomach and its Possible Serious Consequences, Boardman Reed; Goitre-Sporadic Cretinism, Exophthalmic Goitre, Jos. B. Marvin.

NEUROLOGY.

A Case of Hemiplegia, etc., Byrom Branwell; A Case of Electric Chorea, etc., H. A. Hare; A Case of Ischaemic Paralysis, etc., Raymond Johnson; Two Cases of Muscular Dystrophy, George J. Preston; Tubercular Meningitis in Adult, Judson S. Bury; Nature and Treatment of Chorea, etc., F. Warner.

SURGERY.

Causes of Symptoms that Follow Ávacuation of Residual Urine, etc., C. W. Mansell Moullin; Fracture of Lower End of Radius, etc., John B. Roberts; Cleft Palate, T. P. Pick; Potts' Disease of Spine, Jas. K. Young; Hemorrhoids, etc., John B. Hamilton; Tuberculous Bone Disease, W. L. Rodman; Circumcision, etc., H. H. Grant; Operation for Movable Kidney, W. H. Wathen; Perineal Urethrostomy by Poucet's Method, C. G. Cumston; Popliteal Aneurism, etc., A. E. Halstead.

GYNECOLOGY AND OBSTETRICS.

Tuberculosis of Breast, etc., E. P. Davis; Abdominal Palpatism in Pregnancy, A. H. F. Barbour; Sloughing Fibroid, G. E. Montgomery; Sterility in the Female, A. Morgan Cartledge.

OPHTHALMOLOGY.

Diagnosis of Iritis, H. F. Hansell.

LARYNGOLOGY.

Faculty of Speech, etc., G. H. Makuen; Diseases Martoid, S. S. Bishop; Influence of Blake Paper Disk, etc., R. Barclay.

DERMATOLOGY.

Macular Leprosy, A. H. Ohmann Dumesnil; Tinea Circinata, etc., A. Abbott Cambrell.

